# Factors associated with early initiation of breastfeeding in Guinea: Analysis DHS 2018 and implications for public health

**DOI:** 10.4102/jphia.v16i1.1449

**Published:** 2025-10-31

**Authors:** Fanta Barry, Jérôme W. Some, Ramata Diallo, Kaba S. Keita, Madeleine Touré, Tiany Sidibé, Sadan Camara, Aissatou Diallo, Hermann B. Lanou, Alpha O. Sall, Mamadou D. Baldé, Alexandre Delamou

**Affiliations:** 1 Department of Public Health, Centre for Research in Reproductive Health in Guinea (CERREGUI), Conakry, Guinea; 2 Department of Public Health, Health Sciences Research Institute of Burkina Faso (IRSS), Ouagadougou, Burkina Faso; 3 African Centre of Excellence for Prevention and Control of Transmissible Diseases (CEA-PCMT), University Gamal Abdel Nasser, Conakry, Guinea; 4 Department of Public Health, Maferinyah National Centre for Training and Research in Rural Health (CNFRSR), Conakry, Guinea

**Keywords:** associated factors, early initiation of breastfeeding, multilevel analysis, DHS, Guinea

## Abstract

**Background:**

Early initiation of breastfeeding is crucial for the survival of newborns, as it significantly reduces infant mortality rates. However, in Guinea, this practice remains below the targets set by the World Health Organization, which stated that each member country should achieve a rate of early initiation of breastfeeding of 70% by 2030.

**Aim:**

This study aims to identify the factors associated with the early initiation of breastfeeding in Guinea.

**Setting:**

This study was conducted in Guinea.

**Methods:**

Multilevel logistic regression was carried out to identify the determinants of early initiation of breastfeeding. Three two-level statistical models were adjusted and the final model was obtained using a stepwise backwards approach.

**Results:**

Only 42.8% (95% confidence interval [CI]: 39.7–46.0) of mothers reported having breastfed their newborn babies within the first hour of life. Factors associated with early initiation of breastfeeding included caesarean delivery (adjusted odds ratio [aOR] = 0.29; 95% CI: 0.16–0.53, *p* < 0.001), assistance at delivery (aOR = 1.62; 95% CI: 1.26–2.07, *p* < 0.001), the woman’s employment (aOR = 1.38; 95% CI: 1.11–1.73, *p* < 0.003), small birth size (aOR = 0.67; 95% CI: 0.48–0.94, *p* < 0.023) and belonging to rich and very rich households (aOR = 1.60; 95% CI: 1.11–2.31, *p* < 0.011 and aOR = 2.05; 95% CI: 1.33–3.17, *p* < 0.001).

**Conclusion:**

Less than half of women in Guinea initiate breastfeeding early.

**Contribution:**

These results underline the importance of strengthening prenatal care and awareness-raising interventions to improve breastfeeding practices in Guinea.

## Introduction

Malnutrition is a major cause of infant mortality, responsible for 2.7 million deaths annually among children under the age of five, accounting for 45% of all child deaths.^1^ In 2017, approximately 78 million newborn babies were breastfed more than 1 h after birth.^2^ However, immediate initiation of breastfeeding is crucial for the child’s survival.^3^

Scientific evidence indicates that early initiation of breastfeeding reduces neonatal infections and the mortality rate among newborns (42%).^4^ In addition, this practice offers numerous benefits for both the mother and the child: it stimulates breast milk production and reduces the risks of breast and ovarian cancer, cardiovascular disease, postpartum haemorrhage, and type 2 diabetes in mothers.^1,5,6,7^

According to the World Health Organization (WHO) and United Nations Children’s Fund (UNICEF), adequate breastfeeding up to the age of two could save more than 820 000 children under the age of five each year.^8^ Globally, during the period from 2016 to 2022, the rate of breastfeeding newborns within 1 h of birth was 46%, which is lower than the WHO global target of 70%.^9^ In sub-Saharan Africa, this rate varies by region: 61% in Kenya in 2024, 86.3% in Rwanda in 2023, 68% in Uganda in 2020, 50.6% in West Africa in 2024 and 63.4% in Burkina Faso in 2020.^10,11,12,13,14^ Studies in Sudan, Ethiopia and Malawi have identified factors associated with early initiation, but few have explored contextual factors.^15,16,17^

In Guinea, the 2022 Standardised Monitoring and Assessment of Relief and Transitions (SMART) national nutrition survey revealed a low rate of early breastfeeding initiation at only 25.8%.^18^ This is because some communities use the Quranic verse water as their first food, believing that it protects the child, without considering the consequences for the health of the newborn. To achieve the WHO goals, the Guinean government has adopted measures to promote breastfeeding, including infant and young child feeding (IYCF).^19^ Despite these efforts, progress remains limited, especially for early initiation, highlighting the need for contextual studies to understand the factors influencing this practice and better guide policies and programmes.

In Guinea, research focuses primarily on exclusive breastfeeding, leaving a gap in the factors associated with early initiation.^20^ This study aims to fill this knowledge gap by identifying these factors to improve breastfeeding rates and achieve the new goals set for 2030.

## Research methods and design

### Study framework

The Republic of Guinea is a country located in West Africa. It had a population of 14 million according to projections for 2025.^21^ Approximately 43% of the population lived below the poverty line, estimated at GNF 16 423/person per day or 1.6 euros in 2020.^22^ The Guinean households are unevenly distributed, with nearly two-thirds of households located in rural areas (65%) compared to 35% in urban areas. The Total Fertility Rate indicates an average of 4.8 children per woman in 2018.^23^ Guinea faces enormous maternal and neonatal health problems in a context of persistent poverty and periodic health crises. The maternal mortality rate in 2020 was 553.4 deaths per 100 000 live births and a neonatal mortality rate of approximately 31.0 deaths per 1000 live births.^24^ The Guinean primary health care system is supported at the grassroots level by community health workers (CHWs) who provide health education, promote awareness-raising, and facilitate prevention and referral of women for maternal healthcare in health facilities.^25^

### Study type and period

This is a secondary analysis of data from the 2018 Demographic and Health Survey (DHS) of Guinea, a cross-sectional survey. This study used data from the women’s individual questionnaire, specifically information on children’s nutritional practices, including breastfeeding.

### Study population and sampling

The study population included women aged from 15 years to 49 years who participated in the survey. Among the 7951 women surveyed, 3066 were included in this analysis because their children were born within the 24 months preceding the survey and had been breastfed. Women whose children were older than 24 months were excluded. The DHS used a two-stage stratified sampling design, representative at the national level and by place of residence, taking into account urban and rural clusters. This sampling is described in detail in the 2018 DHS report.^23^

### Study variables

#### The dependent variable

The dependent variable was the early initiation of breastfeeding. This variable was dichotomised as ‘1 (Yes)’ for mothers who responded that they had breastfed their newborn babies immediately or within the first hour of life and ‘0 (No)’ for those who responded that they had started breastfeeding their newborn babies 1 h after birth.^5,26,27^

#### Independent variables

The independent variables at the individual and household levels (Level 1) were the individual characteristics of the mother, the child and the household: the age of the mother (15–24 years, 25–34 years and 35–49 years), educational level of the mother (no education, primary and secondary/higher education), the marital status of the mother (single, married/free union and widow/divorced/separated), the current employment of the mother (no and yes), the parity of the mother (primiparous, pauciparous and multiparous), the assistance at delivery during the child’s birth (unassisted delivery and assisted delivery), the number of prenatal consultations (no antenatal consultation, 1 to 3 antenatal consultations and 4 or more antenatal consultations), the mode of delivery (natural delivery and caesarean section), place of delivery (home and health facility), media exposure (no and yes), household socioeconomic status (very poor, poor, average, rich and very rich), the area of residence (urban and rural), household size (≤ 6 members and > 6 members), the father’s educational level (no education, primary and secondary/higher education), the child’s gender (male and female), the child’s birth status (single birth and multiple birth) and the child’s size at birth (*grande taille* [large size], *taille moyenne* [medium size] *and petite taille* [small size]).^28,29^ Based on the data in the literature, the selection of independent or explanatory variables that are potentially associated with early breastfeeding was carried out. The variables were recoded according to the needs of the analysis. The independent variable at the community level (Level 2) was the enumeration area. The contextual variable in our study was the region of residence (coded 1 = Conakry; 2 = Boke; 3 = Faranah; 4 = Kankan; 5 = Kindia; 6 = Labe; 7 = Mamou, and 8 = N’Zerekore).

### Data processing and analysis

The data were processed and analysed using Stata software version 16.1. For the present article, we used secondary data DHS 2018 Guinea. Outliers were detected by observing the data and processed. Sampling weights were applied to correct for oversampling and undersampling, while the SVY command was used to make modifications to the clustered sample design, weights and standard error calculation. After a descriptive analysis, a univariate analysis estimated the association between early breastfeeding initiation and selected variables at the individual and contextual levels using binary logistic regression. The variables with a *p* < 0.25 were included in the multivariate model. A multilevel random-effect binary logistic regression was carried out using a forward stepwise method. Adjusted odds ratios (aOR) were calculated with their *p*-values and 95% confidence intervals (CI). The models were compared using the likelihood ratio test, and the final model specification was validated using post-estimation tests.

Three statistical models were implemented:

The first model contains no independent variables, allowing us to compare our multilevel regression model with a conventional logistic model and estimate the variance in early breastfeeding initiation, which could be attributed to enumeration areas. The second model includes individual-level explanatory variables. The third, or full model, was adjusted with the individual variables and the contextual variable, which is the region of residence.

The models were compared using the intraclass correlation coefficient, used as a measure of variance (random effects). The model with the smallest variance value was chosen as the best fit. The random effects models allow us to distinguish the variability of early initiation by individual characteristics and the region of residence.

### Ethical considerations

The Demographic and Health Survey (DHS) received approval from the Guinean Health Research Ethics Committee. The protocols and questionnaires were reviewed and approved by the Institutional Review Committee of the International Coach Federation (ICF) and the National Health Research Ethics Committee (CNERS) of Guinea. All participants provided informed consent, with parental and/or guardian consent for adolescents 30 years. Data collection and processing ensured privacy and confidentiality. Permission to use the analysed database was obtained following an approved request from the DHS Program (https://dhsprogra.com).

## Results

### Sample characteristics

The mean age of the mothers was 29.1 ± 7.3 years; those aged 25 years to 34 years constituted the most represented age group (45.4%) (Appendix 1, Table 1-A1). Almost all mothers were married (94%), and almost three-quarters of them resided in rural areas (71.4%). Just under a quarter of the mothers lived in households considered very poor (23.6%). Half (51.4%) of the children were perceived as size at birth.

### Rate of early breastfeeding initiation in Guinea

The study revealed that only 42.8% (95% CI: 39.7–46.0) of mothers practised early breastfeeding initiation in Guinea.

### Associated factors of early breastfeeding initiation

Associated factors included mode of delivery, delivery assistance, mother’s labor, perceived size of the baby at birth, and household socioeconomic status (Table 2-A1).

In the multilevel analysis, variables such as the mother’s and father’s level of education, the number of prenatal visits, and parity were not significantly associated with early initiation of breastfeeding in Guinea at the 5% threshold.

The odds of initiating early breastfeeding were 71% less likely (aOR = 0.29, 95% CI: 0.15–0.54, *p* < 0.001) among mothers who delivered by caesarean section as compared to those who delivered vaginally. Similarly, the odds of putting newborns to the breast within the first hour of life were 1.62 times (aOR = 1.62, 95% CI: 1.26–2.07, *p* < 0.001) higher among mothers who received assistance from a qualified health care professional during delivery than among their counterparts who gave birth with no assistance. Mothers reporting labour at the time of the survey were 38% more likely to put their newborns to the breast within 1 h of birth, compared with those not in labour (aOR = 1.38, 95% CI: 1.11–1.73, *p* < 0.003). Similarly, the probability of early breastfeeding was 33% lower (aOR = 0.67, 95% CI: 0.48–0.94, *p* < 0.023) in low-birth-weight infants than in infants reported as high-birth-weight.

Finally, the probability of early breastfeeding was respectively 1.60 and 2.05 (aOR = 1.60, 95% CI: 1.11–2.31, *p* < 0.011 and aOR=2.05, 95% CI: 1.33–3.17, *p* < 0.001) times higher among mothers from rich and very rich households than among those from very poor households.

In the empty model, 33% of the variance in early breastfeeding initiation was attributable to enumeration areas. After the inclusion of individual and contextual variables, this variance decreased to 24%. This reduction indicates that early breastfeeding initiation is influenced by the individual, household and regional characteristics.

## Discussion

The results of the study reveal that in 2018, less than half of women in Guinea practised early initiation of breastfeeding. The main factors associated with this practice include the mode of delivery, birth attendance, the mother’s employment, the perception of the child’s birth size and the household socioeconomic status. These findings are crucial for improving maternal and newborn health in Guinea.

The prevalence of early initiation of breastfeeding in Guinea is lower than that observed in Tanzania (2020), Indonesia (2021), Ethiopia (2022) and Mauritania (2024).^5,29,31,32^ Breastfeeding within 1 h of birth in Guinea remains far from the 70% threshold set by the WHO to be achieved by countries by 2030. This low prevalence could be explained by the high rate of deliveries without skilled attendance (40.5% in this study). Studies in Nigeria and Nepal show that mothers giving birth in health facilities are more likely to practise early breastfeeding.^5,15,27,33^ A low initiation rate could increase neonatal morbidity and mortality. It is therefore crucial to increase awareness-raising campaigns among pregnant women to attend prenatal consultations, where they can receive advice on the benefits of early breastfeeding, which could reduce the influence of cultural beliefs.

Women who delivered by caesarean section were less likely to practise early breastfeeding. This finding is consistent with studies conducted in Ethiopia, Malawi, Sudan, Tanzania and Saudi Arabia.^5,15,17,31,34^ Anaesthetic effects and postoperative pain may prevent these mothers from initiating breastfeeding early.^5,35,36^ To improve this practice, health providers should educate caesarean mothers about the importance of early breastfeeding, both for the well-being of the newborn and the mother.

Delivery attended by skilled health personnel promotes early breastfeeding. A study in Nepal showed that mothers receiving direct counselling in health facilities were more likely to initiate breastfeeding early.^37^ The immediate skin-to-skin contact after birth may also encourage this practice. To promote early breastfeeding, the Guinean government should expand training for health workers on breastfeeding protection, promotion and support.

The employed mothers were more likely to practise early breastfeeding, in contrast to findings observed in the Middle East, Nigeria and Mauritania.^27,29,38^ In Guinea, these mothers may have the income to access antenatal services, where they receive counselling on the benefits of early breastfeeding.

A negative association has been found between small birth size and early breastfeeding initiation. A study in Nigeria also reported that these children were less likely to be breastfed early.^27^ This could be because small newborn babies are not physically ready to seek the breast, thus limiting their breastfeeding abilities within the first hour, unlike a study in Chad, which showed a higher likelihood of early breastfeeding for these children.^39^ Increased support from families, health personnel and CHWs is essential to provide children with a good start in life.

Mothers from wealthy and very wealthy households were more likely to initiate breastfeeding early. Similar results were observed in Ethiopia, Gambia and Mauritania.^16,29,40^ However, a study in Nepal showed that mothers without financial resources to purchase breastmilk substitutes turned to breastfeeding as their only option.^37^ A study in Sudan did not find an association between early initiation and socioeconomic status.^15^ In Guinea, wealthy mothers have easier access to antenatal services and information on the importance of early breastfeeding. They also have more access to media (radio, television, internet).^41^

### Strengths and limitations

This study is based on representative data from a national survey of the female population of reproductive age in Guinea. However, it has certain limitations. Information on early breastfeeding initiation relied on maternal recall, which may introduce recall bias. Furthermore, by analysing secondary data, we were unable to explore certain influential variables, such as cultural norms, grandmothers’ attitudes, or women’s perceptions of early breastfeeding. The cross-sectional nature of the survey also limits the establishment of temporal relationships between associated factors and breastfeeding initiation. Despite these limitations, the study remains valuable in providing significant insight into the situation in Guinea, thus justifying its publication.

### Implications for research and practice

The results of this study highlight the need for targeted interventions to improve early breastfeeding initiation in Guinea. It is imperative to increase access to prenatal care and educate mothers about the benefits of immediate breastfeeding. Health providers must be trained to better support and promote breastfeeding, especially among mothers who have undergone a caesarean delivery. At the same time, qualitative research is needed to explore cultural and social influences on breastfeeding practices. Such studies can provide valuable information for adapting interventions to local contexts. Finally, policies aimed at reducing poverty can facilitate access to the resources needed for effective breastfeeding. By combining these approaches, progress can be made toward international goals and improve the health of both mothers and newborns.

## Conclusion

The early initiation of breastfeeding remains low in Guinea, failing to meet the WHO goals for 2030. To achieve this, it is essential to improve access to prenatal care and raise awareness among mothers about the importance of this practice. Influential factors include the mode of delivery, birth attendance, the mother’s employment, the perception of the size of the newborn and the socioeconomic status. It is crucial to improve access to health services during pregnancy and delivery, while educating mothers about adequate nutrition. Implementing policies to reduce poverty is also necessary. These findings highlight the importance of strengthening prenatal interventions and raising awareness to improve breastfeeding practices in Guinea, thereby contributing to maternal and infant health.

## Appendix 1

**TABLE 1-A1:** Sociodemographic and economic characteristics of the sample of the study on the determinants of early initiation of breastfeeding in Guinea according to the 2018 Demographic and Health Survey (weighted).

Variables	Number of respondents	Percentage
**Age group (years)**		
15–24	1040	34.3
25–34	1397	45.4
35–49	629	20.2
**Mother’s educational level**		
No Education	2275	74.0
Primary	379	12.2
Secondary/Higher Education	412	13.7
**Father’s educational level**		
No Education	2061	71.0
Primary	224	7.6
Secondary/Higher Education	601	21.2
**Mother’s marital status**		
Single	126	04.2
Married/Free union	2887	94.0
Widow/Divorced/Separated	53	01.7
**Woman currently working**		
No	1051	33.3
Yes	2015	66.6
**Parity**		
Primiparous	583	19.2
Pauciparous	1132	37.5
Multiparous	1351	43.2
**Number of Antenatal consultations**		
No Antenatal Consultation	386	12.9
1 to 3 Antenatal Consultations	1512	50.3
4 Antenatal Consultations and more	1085	36.7
**Place of delivery**		
Home	1290	40.6
Health facility	1776	59.3
**Method of delivery**		
Natural delivery	2959	97.0
Caesarean section	90	02.9
**Assisted delivery**		
Unassisted delivery	1198	40.5
Assisted delivery	1691	59.4
**Media exposure**		
No	1243	40.5
Yes	1823	59.5
**Birth status**		
Single birth	2995	97.7
Multiple birth	71	02.2
**Household size**		
≤ 6 members	1289	42.5
> 6 members	1777	57.4
**Child’s gender**		
Male	1572	51.2
Female	1494	48.7
**Child’s size at birth**		
Large size	1548	51.4
Average size	1148	37.7
Small size	329	10.8
**Household wealth quintile**		
Very poor	747	23.6
Poor	676	22.8
Average	605	19.4
Rich	584	18.5
Very rich	454	15.5
**Region of residence**		
Conakry	252	10.9
Boke	427	10.4
Faranah	432	11.1
Kankan	511	18.8
Kindia	411	15.0
Labe	386	11.9
Mamou	291	07.2
N’Zerekore	356	14.4
**Area of residence**		
Urban	866	28.5
Rural	2200	71.4

Note: Mothers’ average age = 29.1 ± 7.3.

**TABLE 2-A1:** Factors associated with the early initiation of breastfeeding in the Republic of Guinea in 2018 (multilevel analysis).

Variables	Empty model		Model I		Model II		
	Adjusted OR	95% CI	Adjusted OR	95% CI	Adjusted OR	95% CI	*p*
Fixed effects							
Assisted delivery	-	-	-	-	-	-	-
Unassisted delivery	-	-	1.00	-	1.00	-	-
Assisted delivery	-	-	1.63	1.28–2.09	**1.62**	**1.26–2.07**	**0.001**
**Number of prenatal consultations**							
No Antenatal Consultation	-	-	1.00	-	1.00	-	-
1 to 3 Antenatal Consultations	-	-	0.73	0.52–1.03	0. 74	0.52–1.04	0.086
4 Antenatal Consultations and more	-	-	0.91	0.63–1.32	0.92	0.63–1.34	0.687
**Household wealth quintile**							
Very poor	-	-	1.00	-	1.00	-	-
Poor	-	-	1.09	0.81–1.48	1.09	0.81–1.48	0.537
Average	-	-	1.25	0.90–1.72	1.26	0.91–1.75	0.147
Rich	-	-	1.56	1.08–2.25	**1.60**	**1.11–2.31**	**0.011**
Very rich	-	-	1.98	1.27–3.07	**2.05**	**1.33–3.17**	**0.001**
**Perceived size of child at birth**							
Large size	-	-	1.00	-	1.00	-	-
Average size	-	-	0.92	0.74–1.13	0.91	0.74–1.12	0.404
Small size	-	-	0.66	0.47–0.93	**0.67**	**0.48–0.94**	**0.023**
**Woman currently working**							
No	-	-	1.00	-	1.00	-	-
Yes	-	-	1.40	1.12–1.74	**1.38**	**1.11–1.73**	**0.003**
**Mother’s educational level**							
No education	-	-	1.00	-	1.00	-	-
Primary	-	-	0.77	0.56–1.05	0.76	0.56–1.04	0.098
Secondary/Higher education	-	-	0.85	0.60–1.19	0.85	0.60–1.20	0.379
**Mode of delivery**							
Natural delivery	-	-	1.00	-	1.00	-	-
Caesarean section	-	-	0.28	0.15–0.53	**0.29**	**0.15–0.54**	**0.001**
**Father’s educational level**							
No education	-	-	1.00	-	1.00	-	-
Primary	-	-	1.04	0.72–1.50	1. 03	0.71–1.49	0.851
Secondary/Higher education	-	-	1. 01	0.77–1.34	1. 01	0.76–1.33	0.943
**Parity**							
Primiparous	-	-	1.00	-	1.00	-	-
Pauciparous	-	-	1.24	0.93–1.65	1.24	0.93–1.65	0.137
Multiparous	-	-	0.98	0.74–1.31	0.99	0.74–1.31	0.946

Note: Parameters (Random Effects) – Variance Region of Residence: Empty model – adjusted OR =1.67; Model I – adjusted OR = 1.53, 95% CI: 1.14–2.06; Model II – adjusted OR =1.07, 95% CI: 0.59–1.94. Intraclass Correlation: Empty model – *n* = 0.33, 33%; Model I – *n* = 0.31, 31%; Model II – *n* = 0.24; 24%. Bolded data identifies significant values.

OR, odds ratio; CI, confidence interval.
